# RNA sequencing data describing transcriptional changes in aorta of ApoE-/- mice after alpha 7 nicotinic acetylcholine receptor (α7nAChR) stimulation

**DOI:** 10.1016/j.dib.2020.105415

**Published:** 2020-03-12

**Authors:** Marcus A. Ulleryd, Filip Mjörnstedt, Dimitra Panagaki, Li Jin Yang, Kajsa Engevall, Saray Gutierrez, Yixin Wang, Li-Ming Gan, Holger Nilsson, Erik Michaëlsson, Maria E. Johansson

**Affiliations:** a Department of Physiology, Institute of Neuroscience and Physiology, The Sahlgrenska Academy, University of Gothenburg, Gothenburg, Sweden; b Crown Bioscience Inc., Shanghai, China; c Department of Molecular and Clinical Medicine, Institute of Medicine, The Sahlgrenska Academy, University of Gothenburg, Gothenburg, Sweden; d Bioscience Heart Failure, Cardiovascular, Renal and Metabolism, IMED Biotech Unit, AstraZeneca, Gothenburg, Sweden

**Keywords:** Alpha 7 nicotinic acetylcholine receptor, α7nAChR, Chrna7, α7nAChR agonists, Cholinergic signaling, Atherosclerosis, Cardiovascular disease, RNA sequencing

## Abstract

This manuscript is a companion paper to Ulleryd M.U. et al., *“Stimulation of alpha 7 nicotinic acetylcholine receptor (α7nAChR) inhibits atherosclerosis via immunomodulatory effects on myeloid cells”* Atherosclerosis, 2019 [1]. Data shown here include RNA sequencing data from whole aorta of ApoE-/- mice fed high fat diet and treated with the alpha 7 nicotinic acetylcholine receptor (α7nAChR) agonist AZ6983 for 8 weeks using subcutaneously implanted osmotic minipumps. Here we present the top gene networks affected by treatment with AZ6983, as well as the up- and down-regulated genes in aorta after treatment. Further, a URL link to the RNA sequencing datasets submitted to GEO is included.

Specifications table

**Table:** 

Subject	Medicine
Specific subject area	Physiology, Experimental atherosclerosis
Type of data	Table Figure
How data were acquired	RNA sequencing (Nextseq500)
Data format	Raw Analyzed
Parameters for data collection	ApoE-/- mice were treated with alpha 7 nicotinic acetylcholine receptor (α7nAChR) agonist AZ6983 or vehicle for 8 weeks using subcutaneously implanted osmotic minipumps. RNA from whole aorta were extracted and used for RNA sequencing analysis. n=6 per group.
Description of data collection	Data shown here includes top gene networks affected by treatment with AZ6983, identified with IPA software, and a table with Complete list of up- and down-regulated genes in the aorta after treatment with AZ6983, ranked by q-value. We also supply a URL link to the RNAseq datasets submitted to GEO. GEO accession numbers: GSE131162, https://www.ncbi.nlm.nih.gov/geo/query/acc.cgi?acc=GSE131162
Data source location	Gothenburg, Sweden
Data accessibility	Repository name: NCBI (http://www.ncbi.nlm.nih.gov.geo/) Data identification number: GSE131162 Direct URL to data: https://www.ncbi.nlm.nih.gov/geo/query/acc.cgi?acc=GSE131162
Related research article	Ulleryd, M.A., Mjörnstedt, F. , Panagaki, D., Yang, L.J., Engevall, K., Gutierrez, S., Wang, Y., Gan, L., Nilsson, H., Michaelsson, E., Johansson, M., E Stimulation of alpha 7 nicotinic acetylcholine receptor (α7nAChR) inhibits atherosclerosis via immunomodulatory effects on myeloid cells. Atherosclerosis 2019 Aug;287:122–133 PMID: 31260875 DOI: 10.1016/j.atherosclerosis.2019.06.903

## Value of the data

• These data provide information on the transcriptional effects on whole aorta after treatment with alpha 7 nicotinic acetylcholine receptor (α7nAChR) agonist AZ6983 in the atherosclerosis-prone ApoE-/- mouse.
• Researchers interested in atherosclerosis, as well as, α7nAChR signaling will find these data a valuable resource.
• The information provided here may be used for future studies on how α7nAChR stimulation influence the vascular transcriptome.
• These data can generate hypothesis for new studies investigating the α7nAChR-related transcriptomic profiles, as well as signaling pathways, in other tissues
• The present data on α7nAChR signaling is predominantly available from cell culture experiments, using cell lines, this data set provides additional information on the signaling pathways in tissue from long-term treatment in vivo.

## 1 Data description

**Fig. 1:**
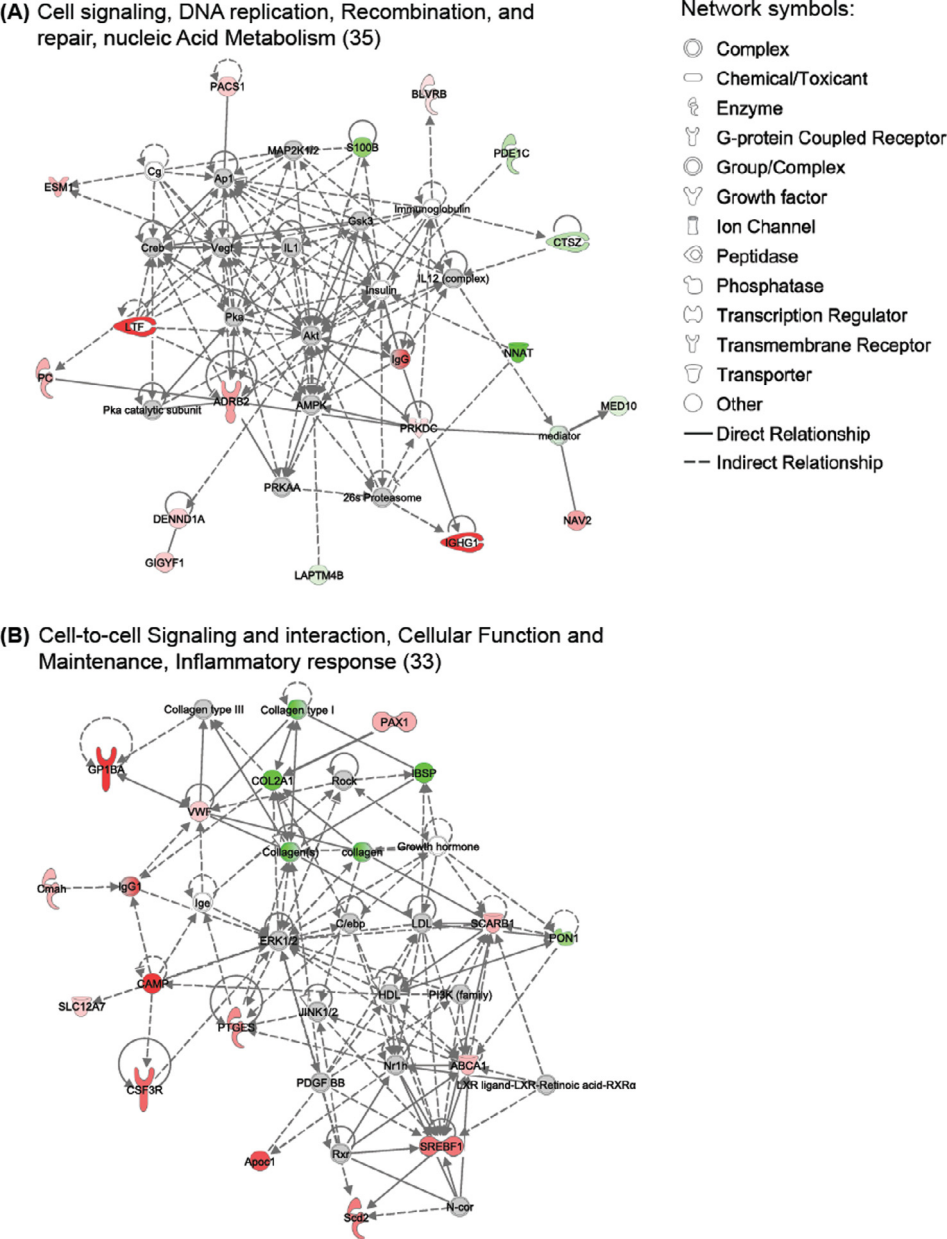
Top gene-networks affected by treatment with AZ6983.

To investigate the effects of alpha 7 nicotinic acetylcholine receptor (α7nAChR) stimulation on atherosclerosis in apolipoprotein E deficient (ApoE-/-) mice, mice were treated with α7nAChR agonist AZ6983 for 8 weeks. Thoracic aortas were used for RNA sequencing analysis. Fig. 1 describes the top two networks identified with Ingenuity Pathway Analysis (IPA) software for differently expressed genes in aorta of ApoE-/- mice treated with AZ6983 compared with controls. Major functions of the networks are indicated in A and B, followed by the network score. Networks are ranked according to their degree of relevance to the eligible network molecules in the data set and the score is calculated with an algorithm based on p-scores derived from q-values. Table 1 shows the complete list of up- and down-regulated genes in the aorta after treatment with AZ6983 ranked by q-value.

**Table 1:** Complete list of up- and down-regulated genes in the aorta after treatment with AZ6983, ranked by q-value.

Symbol	Entrez Gene Name	Up/down	Expr Log Ratio	Expr p-value	Location	Type(s)
IMPDH1	inosine monophosphate dehydrogenase 1	Up	1.427	3.24E−07	Cytoplasm	enzyme
SREBF1	sterol regulatory element binding transcription factor 1	Up	1.010	6.13E−07	Nucleus	transcription regulator
LPCAT3	lysophosphatidylcholine acyltransferase 3	Up	0.594	2.36E−04	Plasma Membrane	enzyme
Scd2	stearoyl-Coenzyme A desaturase 2	Up	0.962	1.22E−03	Cytoplasm	enzyme
MBD6	methyl-CpG binding domain protein 6	Up	0.779	5.23E−03	Nucleus	other
LTF	lactotransferrin	Up	1.647	6.54E−03	Extracellular Space	peptidase
SLC22A23	solute carrier family 22 member 23	Up	0.674	7.01E−03	Other	transporter
Apoc1	apolipoprotein C-I	Up	1.295	7.78E−03	Extracellular Space	other
PXN	paxillin	Up	0.240	7.78E−03	Cytoplasm	other
UTP14C	UTP14C. small subunit processome component	Up	0.665	7.78E−03	Nucleus	other
IGHG1	immunoglobulin heavy constant gamma 1 (G1m marker)	Up	3.965	8.95E−03	Extracellular Space	peptidase
Cmah	cytidine monophospho-N-acetylneuraminic acid hydroxylase	Up	0.551	1.36E−02	Cytoplasm	enzyme
SPON2	spondin 2	Up	0.979	2.09E−02	Extracellular Space	other
Ngp	neutrophilic granule protein	Up	1.934	2.68E−02	Extracellular Space	other
PTGES	prostaglandin E synthase	Up	0.857	3.03E−02	Cytoplasm	enzyme
ABCA1	ATP binding cassette subfamily A member 1	Up	0.514	3.16E−02	Plasma Membrane	transporter
Scd4	stearoyl-coenzyme A desaturase 4	Up	0.902	3.62E−02	Cytoplasm	enzyme
GP1BA	glycoprotein Ib platelet alpha subunit	Up	2.025	4.58E−02	Plasma Membrane	transmembrane receptor
CSF3R	colony stimulating factor 3 receptor	Up	1.122	4.72E−02	Plasma Membrane	transmembrane receptor
H2-M1/H2-M9	histocompatibility 2. M region locus 1	Up	0.950	4.72E−02	Other	other
ADRB2	adrenoceptor beta 2	Up	0.711	4.87E−02	Plasma Membrane	G-protein coupled receptor
MXD1	MAX dimerization protein 1	Up	0.314	5.85E−02	Nucleus	transcription regulator
NPAS2	neuronal PAS domain protein 2	Up	0.780	5.89E−02	Nucleus	transcription regulator
SYTL1	synaptotagmin like 1	Up	1.214	5.89E−02	Cytoplasm	enzyme
LENG8	leukocyte receptor cluster member 8	Up	0.365	6.06E−02	Other	other
WNT2	Wnt family member 2	Up	0.656	6.66E−02	Extracellular Space	cytokine
NAV2	neuron navigator 2	Up	0.667	6.73E−02	Nucleus	other
STIL	STIL. centriolar assembly protein	Up	1.388	6.73E−02	Nucleus	other
ESM1	endothelial cell specific molecule 1	Up	0.560	6.86E−02	Extracellular Space	growth factor
CD177	CD177 molecule	Up	1.525	6.94E−02	Cytoplasm	other
CLK1	CDC like kinase 1	Up	0.297	6.94E−02	Nucleus	kinase
PABPC1	poly(A) binding protein cytoplasmic 1	Up	0.345	6.94E−02	Cytoplasm	translation regulator
PITPNM1	phosphatidylinositol transfer protein membrane associated 1	Up	0.187	7.12E−02	Cytoplasm	transporter
MGAM	maltase-glucoamylase	Up	1.396	7.24E−02	Plasma Membrane	enzyme
PGLYRP1	peptidoglycan recognition protein 1	Up	1.271	7.24E−02	Plasma Membrane	transmembrane receptor
DENND1A	DENN domain containing 1A	Up	0.330	7.26E−02	Plasma Membrane	other
Stfa2/Stfa2l1	stefin A2	Up	2.402	7.26E−02	Cytoplasm	other
BLVRB	biliverdin reductase B	Up	0.183	7.67E−02	Cytoplasm	enzyme
PAX1	paired box 1	Up	0.593	7.74E−02	Nucleus	transcription regulator
PC	pyruvate carboxylase	Up	0.601	7.85E-02	Cytoplasm	enzyme
CYP26B1	cytochrome P450 family 26 subfamily B member 1	Up	0.482	8.03E−02	Cytoplasm	enzyme
Acaa1b	acetyl-Coenzyme A acyltransferase 1B	Up	0.708	8.11E−02	Other	enzyme
CAMP	cathelicidin antimicrobial peptide	Up	2.056	8.11E−02	Cytoplasm	other
SCARB1	scavenger receptor class B member 1	Up	0.573	8.11E−02	Plasma Membrane	transporter
Ifitm6	interferon induced transmembrane protein 6	Up	0.923	8.27E−02	Other	other
VWF	von Willebrand factor	Up	0.369	8.27E−02	Extracellular Space	other
PACS1	phosphofurin acidic cluster sorting protein 1	Up	0.382	8.34E−02	Cytoplasm	other
SLC12A7	solute carrier family 12 member 7	Up	0.386	8.34E−02	Plasma Membrane	transporter
KLHL4	kelch like family member 4	Up	0.442	9.12E−02	Cytoplasm	other
GIGYF1	GRB10 interacting GYF protein 1	Up	0.392	9.62E−02	Extracellular Space	other
DENND2D	DENN domain containing 2D	Up	0.819	9.78E−02	Cytoplasm	other
PRKDC	protein kinase. DNA-activated. catalytic polypeptide	Up	0.259	9.92E−02	Nucleus	kinase
GGACT	gamma-glutamylamine cyclotransferase	Down	−0.361	2.36E−04	Cytoplasm	enzyme
NNAT	neuronatin	Down	−1.894	2.36E−04	Plasma Membrane	transporter
ATP6V1C1	ATPase H+ transporting V1 subunit C1	Down	−0.248	1.33E−03	Cytoplasm	transporter
COL2A1	collagen type II alpha 1 chain	Down	−4.419	6.54E−03	Extracellular Space	other
2210407C18Rik	RIKEN cDNA 2210407C18 gene	Down	−0.642	9.13E−03	Other	other
IBSP	integrin binding sialoprotein	Down	−3.005	2.01E−02	Extracellular Space	other
OTUD6B	OTU domain containing 6B	Down	−0.266	2.68E−02	Other	other
PDE1C	phosphodiesterase 1C	Down	−0.464	4.62E−02	Cytoplasm	enzyme
TCAP	titin-cap	Down	−0.747	4.65E−02	Cytoplasm	other
HEPHL1	hephaestin like 1	Down	−1.625	5.07E−02	Other	enzyme
NUDT4	nudix hydrolase 4	Down	−0.301	5.07E−02	Cytoplasm	phosphatase
CRISPLD1	cysteine rich secretory protein LCCL domain containing 1	Down	−0.570	6.07E−02	Cytoplasm	other
CLEC3A	C-type lectin domain family 3 member A	Down	−3.948	6.41E−02	Other	other
S100B	S100 calcium binding protein B	Down	−0.853	6.41E−02	Cytoplasm	other
LAPTM4B	lysosomal protein transmembrane 4 beta	Down	−0.233	6.66E−02	Cytoplasm	other
CTSZ	cathepsin Z	Down	−0.475	6.81E−02	Cytoplasm	peptidase
EFR3A	EFR3 homolog A	Down	−0.265	6.84E−02	Plasma Membrane	other
GLDN	gliomedin	Down	−0.553	6.86E−02	Cytoplasm	other
ATP6V1A	ATPase H+ transporting V1 subunit A	Down	−0.228	6.94E−02	Plasma Membrane	transporter
MED10	mediator complex subunit 10	Down	−0.234	6.94E−02	Nucleus	other
PON1	paraoxonase 1	Down	−0.775	6.94E−02	Extracellular Space	phosphatase
HCFC1R1	host cell factor C1 regulator 1	Down	−0.196	8.11E−02	Nucleus	other
MSI2	musashi RNA binding protein 2	Down	−0.154	8.25E−02	Cytoplasm	other
AK4	adenylate kinase 4	Down	−0.559	9.69E−02	Cytoplasm	kinase
DPP10	dipeptidyl peptidase like 10	Down	−0.750	9.92E−02	Extracellular Space	peptidase

All differentially expressed genes, after p-value adjustment (q-values) using Benjamini Hochberg [2] and a FDR-q of 10%, in the aorta of AZ6983 treated mice compared with controls. Genes are sorted by up or down regulation, followed by the adjusted p-value.

## 2 Experimental design, materials, and methods

### 2.1 Experimental animals

Male apoE-/- mice (C57BL/6 background, B6192P2-Apoetm1UncN11, Taconic, Denmark) were kept at the Laboratory for Experimental Biomedicine, Gothenburg, Sweden. At 10 weeks of age, mice were anesthetized using isoflurane and subcutaneously implanted with osmotic minipumps (Alzet model 2004, DURECT Corporation, ALZET Osmotic Pumps, Cupertino, CA, USA) delivering vehicle (28% cyclodextrin in saline), or α7nAChR agonist AZ6983 (50 µmol kg-1 per day) for 8 weeks. Due to the duration of the minipumps, they were replaced after 4 weeks. From 10 weeks of age and throughout the experiment, mice were fed a high fat, cholesterol enriched diet (21% fat, 0.15% cholesterol; R638, Lantmännen, Sweden). All animals were housed at 21–24 °C in a room with 12 h light/ 12 h dark cycle. Water and food were available *ad libitum*. All procedures involving mice were approved by the Regional Animal Ethics Committee at the University of Gothenburg, in accordance with the European Communities Council Directives of 22 September 2010 (2010/63/EU).

### 2.2 RNA isolation, RNA sequencing and ingenuity pathway analysis

RNA of thoracic aorta was extracted by using the RNAeasy^Ⓡ^ Fibrous Tissue Mini Kit (Qiagen GmbH, Hilden, Germany) according to the manufacturer's protocol. Concentration and quality was analyzed using a NanoDrop (NanoDrop Products, DE, US) and electrophoresis (Experion, Bio-Rad Laboratories, CA, USA).

Aortic RNA from mice treated with AZ6983 (n=6) or controls (n=6) was isolated as described above and Stranded Total RNA Sample preparations were performed using the Illumina TrueSeq Stranded Total RNA Sample Preparation Kit with Ribo Sero Gold according to the TruSeq Stranded Total RNA Sample Preparation Guide (15031048 Rev. E). Sequencing of the enriched libraries was performed on Illumina Nextseq500 (2x75bp). The quality of the data was analyzed with FastQC and reads with an average quality score of >30 were included in the sequencing. Differentially expressed genes (DEGs) were identified using the DESeq2-method with Benjamini Hochberg adjusted p-values (q-values) [2] and a FDR-q of 10%.

QIAGEN's Ingenuity^Ⓡ^ Pathway Analysis (IPA^Ⓡ^, QIAGEN Redwood City, content version 42012434) was used to study potential functions of AZ6983 treatment in the aorta [3], [4], [5]. Network analysis was generated by overlaying the eligible network molecules in the data set with the global gene network contained in the Ingenuity^Ⓡ^ Knowledge Base. Networks are ranked according to their degree of relevance to the genes in the data set. Functional analysis identified the top ranked biological functions and diseases that were enriched in the dataset by calculating the number of molecules that cohere to a functional category and was estimated by Fisher's exact test (q<0.05). Activation Z-score predicts if a specific function is activated (≥ 2) or inhibited (≤ −2) and is supported by one or more references from Ingenuity^Ⓡ^ Knowledge Base.

Top two networks identified with Ingenuity Pathway Analysis (IPA) software for genes that were differently expressed in aorta of ApoE^-/-^ mice treated with AZ6983 compared with controls. Major functions of the networks are indicated in A and B, followed by the network score. Networks are ranked according to their degree of relevance to the eligible network molecules in the data set and the score is calculated with an algorithm based on p-scores derived from q-values. The up- (red) or down– (green) regulation of genes are indicated by the intensity of node color, and the functional class of the gene product is indicated by different symbols. Relationship between genes are supported by one or more references and illustrated with a connecting line.

## Appendix Supplementary materials

Image, application 1
